# Oropouche Virus: An Emerging Arboviral Threat and Its Implications for Europe

**DOI:** 10.3390/life15111674

**Published:** 2025-10-27

**Authors:** Gaetano Scotto, Vincenzina Fazio, Salvatore Massa

**Affiliations:** 1 Infectious Diseases Unit, University Hospital “OORR” Foggia, 71122 Foggia, Italy; 2 Clinical Chemistry Laboratory, Virology Unit, University Hospital “OORR” Foggia, 71122 Foggia, Italy; 3 Department of Agriculture, Food, Natural Resource and Engineering, University of Foggia, 71122 Foggia, Italy

**Keywords:** Oropouche virus, arbovirus, vector competence, epidemiology, vertical transmission, Europe surveillance, travel medicine

## Abstract

Oropouche virus (OROV), an emerging arbovirus of the Peribunyaviridae family, is responsible for acute febrile illness and, in some cases, neurological or hemorrhagic complications. Although traditionally confined to tropical areas of Central and South America, the 2024–2025 epidemic has signaled a major shift in its geographic and clinical profile, with sustained transmission in the Caribbean, over 15,000 confirmed cases, and the first imported infections reported in Europe and the United States. New clinical observations include fatalities in previously healthy adults, suspected vertical transmission with adverse fetal outcomes, and potential sexual transmission. Despite entomological data indicating low competence of European mosquito species and the absence of the main vector *Culicoides paraensis*, the increasing frequency of imported cases underscores the need for continued vigilance. Diagnostic limitations and clinical overlap with other arboviruses further complicate early detection. This review summarizes current knowledge on OROV’s epidemiology, transmission dynamics, and clinical features, and highlights the urgent need for integrated surveillance, diagnostic readiness, and coordinated research efforts. Emphasis is placed on Europe’s preparedness strategies, with Italy’s Jubilee 2025 offering a real-world case study for managing arboviral threats during mass gatherings.

## 1. Introduction

Recent decades have witnessed a substantial increase in the global circulation of human arboviruses, particularly within the Togaviridae, Flaviviridae, and Bunyaviricetes families, resulting in the emergence or re-emergence of significant infectious threats including Dengue, Zika, Chikungunya, West Nile virus (WNV), Usutu, and tick-borne encephalitis (TBE) [1,2]. While these pathogens occasionally manifest as sporadic cases, they more frequently precipitate extensive epidemics, particularly throughout tropical and subtropical regions of the Americas [3,4]. These viruses, along with their suitable vectors, are increasingly establishing new endemic foci in previously unaffected territories [5,6]. Multiple environmental and anthropogenic factors drive this geographic expansion, including climate change, widespread deforestation, and uncontrolled urbanization, which collectively facilitate the colonization of new territories by arthropod vectors [7,8]. Additionally, increased human mobility associated with international tourism, religious pilgrimages, and migration to high-risk regions, combined with inadequate awareness of infectious disease risks, significantly contributes to pathogen dispersal [9,10].

Among these emerging threats, Oropouche virus (OROV), despite being isolated several decades ago, has remained relatively understudied compared to other arboviruses. Initially identified in 1955 in Trinidad and Tobago from blood samples obtained from a febrile forest worker [11,12], OROV has subsequently been implicated in over 30 documented epidemics and more than half a million reported cases across Central and South America and the Caribbean region [13]. However, because OROV infection presents with clinical symptoms similar to those of other co-circulating arboviruses—such as dengue (DENV), chikungunya (CHIKV), Zika (ZIKV), and West Nile virus (WNV)—it is often misdiagnosed, and the true number of cases is likely significantly underestimated [14]. In the last 25 years, cases of Oropouche have been identified in many countries, including Argentina, Bolivia, Brazil, Colombia, Ecuador, French Guiana, Panama, and Peru. In February 2024, the Pan American Health Organization/World Health Organization issued an alert regarding an increasing number of human OROV infections [15]. From the start of 2024 until early December, a total of 13,014 confirmed OROV cases were reported across 12 countries in the Americas, with Brazil accounting for the majority (10,940 cases, including two fatalities). Additionally, imported cases linked to travel were identified in the United States (90 cases), Canada (2 cases), and Europe (30 cases). In 2024, the virus was detected in both urban and rural locations where no previous transmission had been recorded. Deaths associated with the infection were documented, along with instances of vertical transmission (from mother to child during pregnancy), which included fetal deaths and congenital abnormalities [16]. This pathogen currently ranks as the second most prevalent arboviral cause of human infection in the Amazon Basin, exceeded only by dengue virus [17]. A recent editorial in The Lancet Infectious Diseases characterized OROV as a “mysterious threat” deserving of enhanced scientific attention [18].

The present comprehensive review aims to provide an updated synthesis of current knowledge on Oropouche virus (OROV), with particular attention to its virological properties, epidemiological trends, and public health implications for Europe and Italy. Specifically, it (i) summarizes recent data on the molecular evolution and reassortment potential of OROV, including insights from the 2024–2025 epidemic; (ii) examines the latest clinical and epidemiological evidence relevant to disease surveillance and diagnosis; and (iii) assesses the risk of OROV introduction and the potential for local transmission in Europe, with a focus on vector competence and preparedness strategies in the context of the ongoing 2025 Jubilee in Italy. This work is intended to support risk assessment and inform public health planning.

## 2. Virological and Genetic Characteristics

The Oropouche virus (OROV; species name Orthobunyavirus oropoucheense) is one of the 138 recognized species classified as negative-sense single-stranded RNA viruses within the Orthobunyavirus genus in the family Peribunyaviridae [19]. This genus represents the largest and most diverse group in the family and includes viruses distributed across tropical, temperate, and even Arctic ecological niches worldwide. Although a wide range of arthropod and vertebrate hosts are described for viruses of the genus, each virus possesses a restricted arthropod and host range, which in turn limits its geographic distribution [20].

**Figure 1:**
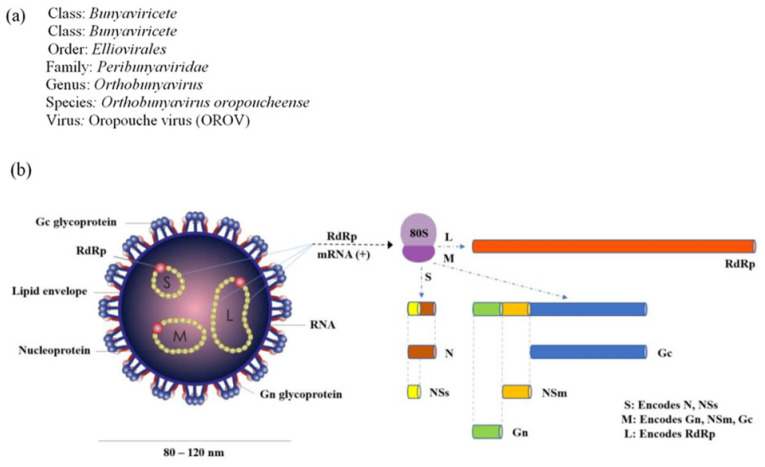
Taxonomical classification (**a**) and schematic of the OROV virion (**b**) (adapted from [12]).

The virion displays a spherical morphology (80–120 nm) with an external lipid envelope acquired from host cells through budding and is embedded with trimeric glycoprotein spikes (Gn and Gc), responsible for receptor binding and membrane fusion (Figure 1). The internal structure includes ribonucleoprotein (RNP) complexes, each composed of one of the three RNA segments (S, M, L) coated with nucleocapsid (N) proteins and associated with the viral RNA polymerase (RdRp). The RNA genome gives rise to six structural and non-structural proteins [21]. Non-structural proteins NSs and NSm, encoded by the S and M segments, respectively, are also indicated for their roles in immune evasion and virion morphogenesis. Matrix proteins lining the inner envelope contribute to virion stability and assembly [22].

The S segment encodes the nucleocapsid (N) protein, which packages the viral RNA, and a non-structural NSs protein that functions as a virulence factor by suppressing host type I interferon responses, thereby facilitating viral replication and immune evasion [22,23]. The M segment encodes two envelope glycoproteins, Gn and Gc, which are essential for viral attachment to host cell receptors and subsequent membrane fusion during cellular entry [24]. The L segment encodes the viral RNA-dependent RNA polymerase (RdRp), which is indispensable for genome replication and mRNA synthesis during the viral life cycle [25]. Phylogenetic analyses based on S-segment RNA sequences have established four primary OROV genotypes (I–IV), with distinct geographic distributions and evolutionary relationships [25].

As a segmented virus, OROV is capable of genetic reassortment during co-infection with related viruses, potentially yielding new progeny with mixed segment origins and altered pathogenicity or transmission dynamics. This mechanism occurs during genome replication, when multiple viral strains simultaneously infect a single cell, enabling segment exchange between different OROV strains or related orthobunyaviruses [26,27]. The tripartite genome structure facilitates the emergence of novel reassortant variants with modified genetic combinations that may exhibit enhanced virulence or improved immune evasion capabilities. Three OROV reassortments have recently been identified in South America: Iquitos virus isolated in Peru, Madre de Dios isolated in Peru and Venezuela (both pathogenic to humans), and Perdoes in Brazil. These viruses share the S and L segments with OROV but have an M segment derived from unidentified orthobunyaviruses. Recent epidemiological evidence demonstrates that such reassortant OROV strains have contributed to widespread outbreaks, with some variants associated with severe clinical outcomes including maternal–fetal infection during pregnancy, stillbirth, congenital microcephaly, and malformation syndrome [28]. The 2024 outbreak notably featured two documented deaths in previously healthy young women, along with multiple reports of pregnancy complications including miscarriage, fetal deaths, and microcephaly associated with OROV infection, highlighting the potential severity of emerging reassortant strains [29].

## 3. Transmission Cycles and Epidemiology

**Figure 2:**
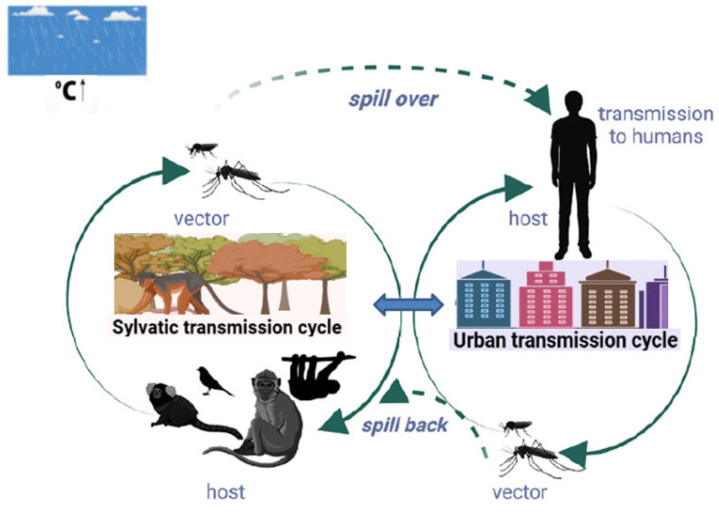
OROV transmission cycles. The sylvatic cycle involves various wild mammals and arthropod vectors, while the urban cycle includes humans and the biting midge *Culicoides paraensis* as the primary vector. Solid lines represent the distinct sylvatic and urban cycles; dotted lines indicate their interconnection. OROV can ‘spill over’ into humans (i.e., transmission from animal reservoirs to humans), triggering the urban cycle, and ‘spill back’ into wildlife and vectors, thus sustaining viral circulation. The cloud and upward arrow next to the temperature symbol (°C ↑) indicate that increased humidity and temperature may favor viral transmission and vector proliferation (adapted from [32]).

OROV transmission to humans occurs through two interconnected but distinct ecological cycles: sylvatic and urban transmission pathways [30,31] (Figure 2).

The sylvatic cycle represents the primary maintenance mechanism for OROV in nature, involving enzootic transmission among wild vertebrate hosts and forest-dwelling arthropod vectors. The key animal host for OROV appears to be the three-toed sloth (*Bradypus tridactylus*). Since insects transmit the virus from sloths to humans and vice versa, it has been nicknamed “sloth fever”. In this cycle, three-toed sloths (*Bradypus* spp.) function as crucial amplifying hosts due to their remarkable susceptibility to infection and capacity for sustained high-level viremia. Additional mammalian hosts include various species of nonhuman primates, rodents, and potentially certain bird species, though the latter’s role in viral maintenance remains incompletely characterized [32].

The urban cycle emerges when OROV transitions from sylvatic environments to human-dominated landscapes, primarily through the activity of *Culicoides paraensis*, an anthropophilic biting midge that exhibits strong preference for human blood meals. This species thrives in peridomestic environments and can establish breeding sites in various organic materials, facilitating sustained transmission within human communities. Secondary urban vectors may include *Culex quinquefasciatus*, which can contribute to transmission under favorable environmental conditions. However experimental data indicate a high infection threshold (≥9.5 log_10_ SMLD_50_/mL) for *Cx. quinquefasciatus*, suggesting this species is an inefficient vector [33]. Several studies have excluded domestic animals such as cats, dogs, and chickens from the urban transmission cycle of OROV, supporting the hypothesis that humans are the only vertebrate hosts. Moreover, there is no evidence of direct human-to-human transmission [32]. Humans are likely the bridge between the sylvatic and urban transmission cycles of OROV, as outbreaks in urban areas often originate from viremic individuals returning from forested regions where they acquired the infection [17]. Nevertheless, important gaps remain in our understanding of the transmission dynamics. For example, the low isolation rate of OROV from midges during epidemics (approximately 1:12,500) raises questions about the vector’s susceptibility to the virus or whether only a subset of the midge population is capable of transmitting OROV. Further research is needed to clarify these aspects [12].

## 4. Clinical Manifestations

Human infections with OROV typically present as an acute febrile illness with sudden onset. The core clinical features include high-grade fever (≈39 °C), severe headache, myalgia, arthralgia, nausea, vomiting, and retro-orbital pain. Additional symptoms commonly reported include photophobia, fatigue, conjunctival hyperemia, anorexia, abdominal pain, and a maculopapular rash [34]. Less frequent manifestations include diarrhea and mild hemorrhagic signs such as epistaxis, gingival bleeding, and petechiae [14,35]. In most cases, the disease follows a self-limiting course lasting 2 to 7 days, with complete recovery and no sequelae. However, up to 60% of patients may experience a biphasic illness characterized by an initial remission followed by a recurrence of symptoms—commonly fever, headache, myalgia, and asthenia—occurring 2 to 10 days after defervescence [36].

Although severe complications remain rare, they are clinically significant, and the 2024 epidemic revealed concerning patterns. Neurological involvement, including aseptic meningitis and meningoencephalitis, has been documented in association with Oropouche virus (OROV) infection, representing some of its most severe clinical manifestations. Although such central nervous system (CNS) complications are relatively rare, OROV has been identified in cerebrospinal fluid (CSF) in confirmed neuroinvasive cases, indicating the virus’s capacity to breach the blood–brain barrier [37]. These conditions tend to occur more frequently in immunocompromised individuals and children, who appear to be at greater risk for severe neurological outcomes [36,38,39]. Cases of Guillain-Barré syndrome and prolonged post-infection asthenia and myalgia have also been documented [40].

Adverse pregnancy outcomes such as miscarriage and congenital anomalies—microcephaly, ventriculomegaly, and agenesis of the corpus callosum—have been linked to vertical transmission of OROV [41]. While historically considered a non-lethal virus, the 2024 Brazilian outbreak marked a turning point, with confirmed fatalities in previously healthy adults who presented with multisystem involvement, including hemorrhagic manifestations, hypotension, respiratory failure, and shock. These events coincided with the emergence of a more virulent OROV strain (AM0088), characterized by higher replication rates in mammalian cells compared to earlier genotypes [42].

A major clinical challenge posed by OROV infection is its significant symptom overlap with other arboviral diseases endemic to the same regions—namely dengue virus (DENV), chikungunya virus (CHIKV), and Zika virus (ZIKV). All four infections commonly present with fever, rash, arthralgia, myalgia, and headache, particularly in the early stages. While each virus has distinguishing features—such as hemorrhagic tendency in DENV, polyarthritis in CHIKV, and neurodevelopmental complications in ZIKV—the initial clinical picture is often nonspecific and can lead to diagnostic confusion [14,34,43]. Moreover, OROV and other arboviruses share similar transmission vectors (e.g., *Culicoides paraensis* midges, Culex and Aedes mosquitoes) and may cross-react in serological tests, complicating laboratory diagnosis. Cases of co-infection with DENV, ZIKV, or CHIKV have also been reported, further obscuring clinical identification and increasing the risk of underdiagnosis, particularly in regions with limited molecular diagnostic capacity [44]. These diagnostic challenges are particularly relevant in non-endemic regions such as Europe, where clinicians may be unfamiliar with the clinical profile of OROV. Increased awareness and the inclusion of OROV in differential diagnoses of acute febrile illnesses—especially in returning travelers from endemic areas—are essential to improve early detection and public health response.

## 5. Diagnosis, Treatment, and Vaccine Development

Laboratory confirmation of OROV infection is essential due to clinical overlap with other arboviral diseases such as dengue, chikungunya, and Zika. Molecular detection through real-time reverse transcription PCR (RT-PCR) serves as the cornerstone of early diagnosis, capable of detecting viral RNA in serum, plasma, saliva, or urine during the first 5–7 days after symptom onset [45,46,47]. RT-PCR assays targeting the M segment offer higher specificity due to reduced susceptibility to genomic reassortments [48].

Serological testing through ELISA or indirect immunofluorescence can detect OROV-specific IgM antibodies during the second week of illness, when viremia has declined [39]. Seroconversion demonstrated by a ≥4-fold rise in antibody titer between acute- and convalescent-phase sera provides definitive confirmation [44]. However, cross-reactivity with other orthobunyaviruses can reduce specificity, and neutralization tests remain the reference standard despite requiring BSL-3 facilities [43]. Currently, no internationally standardized diagnostic protocol or commercial assay exists for OROV, with laboratories using different primer sets and interpretation criteria, highlighting the urgent need for validated, harmonized diagnostic algorithms [49,50].

No specific antiviral therapy is currently approved for OROV infection. Clinical management relies on supportive care including rest, adequate hydration, and the administration of analgesics and antipyretics [40]. Due to potential hemorrhagic manifestations, aspirin and NSAIDs should be avoided to minimize bleeding risk. Experimental investigations have yielded limited results: ribavirin demonstrated activity against related orthobunyaviruses but failed against OROV in vitro and in animal models [51]. Mycophenolic acid similarly proved ineffective [52]. Interferon-α showed dose-dependent antiviral activity in vitro and prophylactic efficacy in mice when administered before infection, though post-infection treatment failed to prevent viral replication or reduce mortality [52]. Favipiravir, a broad-spectrum RNA polymerase inhibitor active against related Peribunyaviridae members, represents a promising candidate awaiting evaluation against OROV [50,53,54].

Vaccine development efforts have advanced several experimental approaches. A live attenuated vaccine based on OROV strain BeAn19991 has demonstrated high neutralizing antibody induction and broad protection across multiple viral strains in animal models [25,55]. Recombinant vesicular stomatitis virus (rVSV) vectors expressing OROV glycoproteins Gn and Gc produced robust neutralizing antibody responses and protected mice from fatal infection in preclinical studies [56]. Computational epitope mapping of the M-segment polyprotein has identified B- and T-cell targets supporting peptide-based or subunit vaccine design [57]. Development of reverse genetics systems enables the creation of attenuated strains with preserved immunogenicity while facilitating pathogenesis studies [22].

However, significant challenges persist, including high genetic variability among circulating strains and reassortant viruses (Iquitos, Madre de Dios, Perdões), raising concerns about cross-protective efficacy. Limited availability of animal models that replicate human infection complicates vaccine evaluation [13,58]. Future progress will likely require combined approaches encompassing live-attenuated, viral-vectored, DNA, and protein-subunit platforms, supported by international collaboration, sustained funding, and continuous epidemiological surveillance to guide vaccine updates.

## 6. Recent Outbreaks: 2024–2025

During 2024 and 2025, Oropouche virus has caused its most widespread epidemic to date, with local outbreaks and imported cases reported across the Americas and beyond. Brazil has been the most affected country, with over 9500 confirmed cases in 2024 and more than 5500 additional cases by mid-2025. While the majority of infections occurred in the Amazon region, the virus has also spread to at least 22 of the 27 Brazilian states, including previously unaffected areas such as Bahia, Espírito Santo, Rio de Janeiro, Minas Gerais, Santa Catarina, and Ceará [16,59]. Two deaths were reported in young adult women in Bahia in 2024 [60]. Laboratory investigations identified a reassortant strain of the virus, possibly contributing to increased transmissibility [60]. Unlike past outbreaks, transmission has persisted across multiple rainy seasons, suggesting changing ecological conditions that favor sustained vector activity [61].

In several countries outside Brazil, OROV circulation has also been confirmed. Cuba experienced its first documented epidemic with over 600 cases in 2024, mostly among women, and associated with neurological complications such as Guillain-Barré syndrome and congenital abnormalities [62]. In Peru, 936 cases were reported, mainly in the Loreto region. Bolivia confirmed 356 cases, especially in La Paz, with co-infections involving dengue virus [63]. Colombia detected 74 infections as part of a febrile illness surveillance study, including two cases in pregnant women [64]. Sporadic cases occurred in Ecuador and Guyana. Panama reported a single case in 2024 and an additional 79 cases in early 2025, indicating ongoing local transmission [63].

International spread has occurred primarily through travel. In 2024, the United States reported 94 imported cases, most from Cuba and Brazil, including two neuroinvasive infections [65]. In the same year, the European Union reported 19 imported cases, mainly in Spain [66] and Italy [67]. Five more cases were confirmed in early 2025 in Germany [68] and France [69]. Canada and the Cayman Islands also documented single imported cases [70]. No local transmission has been identified outside the Americas, likely due to the absence of competent vectors and unsuitable environmental conditions.

Demographic patterns varied between countries. In Colombia, most infections were reported in adolescents aged 10–19 years, while in Bolivia and Peru, adults aged 30–39 years were most affected. In Cuba, about 55% of patients were female, whereas a more equal gender distribution was observed in Bolivia and Peru [70].

## 7. Drivers of the 2024 Epidemic

Multiple interconnected factors have contributed to the unprecedented scale and geographic scope of the 2024 OROV epidemic. Environmental alterations, including climate change, extensive deforestation, urban encroachment into previously undisturbed habitats, and consequent disruption of wildlife behavior patterns, have significantly influenced epidemic dynamics [7,8]. These environmental changes facilitate increased contact between sylvatic transmission cycles and human populations, promoting spillover events.

Viral genetic evolution represents another critical driver of epidemic expansion. OROV’s segmented genome architecture facilitates genetic reassortment events, which may significantly influence viral replication efficiency, transmissibility, and pathogenic potential [28]. Comparative genomic analyses between historical OROV strains and those circulating during the 2024 outbreak have revealed the emergence of novel viral variants containing genetic segments derived from Iquitos virus, a related orthobunyavirus. These reassortant variants demonstrate enhanced replication capacity in mammalian cell cultures, produce higher viral loads in infected hosts, and exhibit increased pathogenic potential compared to historical strains [28]. Clinical manifestations during the 2024 outbreaks have included severe cardiovascular complications such as ventricular tachycardia and persistent tachycardia, fatal outcomes in previously healthy individuals, and neurological involvement in approximately 4% of cases [39,64]. Most notably, on 25 July 2024, two young Brazilian women without pre-existing comorbidities succumbed to OROV-associated illness, marking the first documented fatalities in healthy adults [64].

## 8. Congenital Infections and Vertical/Sexual Transmission

The 2024 OROV epidemic has raised new concerns regarding vertical transmission—a route previously undocumented for this orthobunyavirus. Multiple independent reports suggest that OROV may cross the placental barrier, potentially leading to congenital infections and adverse pregnancy outcomes. The Pan American Health Organization reported 13 fetal deaths, three spontaneous miscarriages, and four congenital malformations potentially associated with maternal OROV infection during the 2024 outbreak in Brazil [29]. In a related serological and molecular investigation, das Neves Martins et al. [71] identified six newborns with microcephaly who tested positive for OROV, including three born during the 2024 epidemic. One of these infants, who died at 47 days of age, showed OROV RNA and antigen in multiple tissues—including the brain, lungs, kidney, and central nervous system—supporting a possible causal link between intrauterine infection and neuropathological damage.

Ribeiro et al. [72] provided a clinical description of three confirmed cases of congenital OROV infection, all born to symptomatic mothers in the Brazilian Amazon. The newborns exhibited severe microcephaly, arthrogryposis, and structural brain abnormalities, as well as chorioretinal scarring, suggesting a teratogenic profile reminiscent of—but distinct from—congenital Zika syndrome. These cases further support the hypothesis that OROV may cause a recognizable congenital syndrome.

Experimental data from a murine pregnancy model support the biological plausibility of vertical transmission. Gunter et al. [73] demonstrated that both prototype and outbreak strains of OROV replicate in maternal tissues, infect the placenta, and disseminate to fetal organs. Human trophoblast-derived cell line assays confirmed OROV tropism for placental tissue, suggesting that vertical transmission may be an inherent feature of OROV pathogenesis.

Although these findings suggest the possibility of transplacental transmission, definitive causal attribution remains limited by small sample sizes, retrospective sampling, and incomplete pathogen screening in some studies. As such, vertical transmission should be considered plausible but not yet conclusively demonstrated. Nevertheless, the accumulating clinical and experimental evidence has prompted public health authorities to act: the U.S. Centers for Disease Control and Prevention now advises pregnant women to avoid non-essential travel to OROV-endemic regions [40].

Recent virological studies have also raised concern about potential sexual transmission. According to the CDC [40] OROV RNA has been detected in semen and vaginal secretions of patients with acute Oropouche fever, and replication-competent virus has been isolated from semen. These findings are similar to what has been observed with other sexually transmissible arboviruses, such as Zika and Ebola viruses. Additional evidence comes from a documented case in Italy, where a traveler returning from Cuba exhibited prolonged viral shedding in semen—up to 58 days after symptom onset—with confirmed detection of replication-competent virus [74]. Similar results have been recently reported by Iglói et al. [75] in a male patient returning to the Netherlands from Cuba in August 2024. Based on these findings, the CDC recommends that infected men use barrier protection or abstain from sexual activity for at least six weeks following symptom resolution [40].

Despite these observations, no epidemiologically confirmed cases of sexual transmission have been reported. The presence of virus in genital secretions represents potential, but not proof, of transmission. Further research is needed to determine the epidemiological relevance and public health impact of this possible transmission route.

## 9. OROV in Europe: Epidemiological Risk and Vector Competence Assessment

The current risk of OROV infection for the European population and travelers is considered low but not negligible. According to the European Centre for Disease Prevention and Control [76] European travelers visiting endemic or epidemic regions—particularly in northern Brazil and the Amazon Basin—may be exposed to a substantially elevated risk if adequate vector-avoidance measures are not implemented. Infection risk varies based on destination, seasonality, and individual behaviors.

Within Europe, the risk of autochthonous OROV transmission remains theoretical. The principal confirmed vector, *Culicoides paraensis*, is not established in European ecosystems, and no secondary transmission events have been reported [77]. While *Culicoides impunctatus*, a widespread species across Europe, has not been implicated in the transmission of human arboviruses, its vector competence for OROV has not yet been systematically assessed [78]. However, given the increasing number of OROV outbreaks in the Americas and the expansion of international travel, the potential for introduction through infected travelers cannot be excluded, thus underscoring the need for continued surveillance and preparedness.

Experimental investigations conducted in Germany evaluated the vector competence of five mosquito species commonly found in European environments: *Culex pipiens*, *C. torrentium*, *Aedes aegypti*, *A. japonicus*, and *A. albopictus*. Under controlled laboratory conditions, only *A. albopictus* demonstrated minimal infection rates at temperatures between 24–27 °C, with no detectable infection occurring at lower temperatures typical of most European climates [79]. Complementary research conducted by Italian investigators under biosafety level 3 containment exposed field-derived populations of *A. albopictus* and *C. pipiens*, collected from Rome, to an infectious OROV strain (1.7 × 10^6^ TCID_50_/mL) under standardized conditions (26 ± 1 °C, 70% relative humidity, 14:10 h light:dark photoperiod) [77]. Engorged females were monitored over a 21-day incubation period to assess infection, dissemination, and transmission potential. Results demonstrated that only 2 of 60 A. albopictus specimens tested positive for viral RNA in body tissues, yielding a cumulative infection rate of 3.3%. Critically, no virus was detected in legs, wings, saliva, or F1 progeny, indicating the absence of viral dissemination beyond the midgut and lack of transmission capability. No infection was observed in *C. pipiens* specimens under identical experimental conditions. These findings suggest the presence of significant midgut-level barriers to OROV infection in both European mosquito species, consistent with previous investigations conducted in the United States and Europe [77,80,81].

Additionally, several experimental studies summarized by Tilston-Lunel et al. [22] provide further insight into OROV vector dynamics. Historical transmission experiments conducted in the 1980s demonstrated that *C. paraensis* is a highly competent vector, capable of transmitting OROV to hamsters 4–12 days after feeding on viremic hosts, with viral titers ranging from 5.3 to 9.9 log_10_ SMLD_50_/mL [82]. Furthermore, *Culicoides sonorensis*—a midge species native to North America and a known vector of the closely related Schmallenberg virus in northern Europe—was shown to be similarly susceptible, with experimental infections yielding mean viral titers of 2.5 × 10^4^ PFU/mL and successful viral dissemination to legs and saliva [83].

Although *C. sonorensis* is not currently present in Europe, its proven competence for OROV raises concerns regarding the potential role of other midge species, including those established in European habitats. This point becomes particularly relevant considering the spread of invasive species and climate-driven ecological shifts. Meanwhile, laboratory assessments of mosquito species such as *Aedes aegypti*, *A. albopictus*, *Culex quinquefasciatus*, and *C. tarsalis* indicate that these species are only susceptible to OROV infection via direct intrathoracic inoculation, with oral infection rarely resulting in dissemination—again suggesting strong midgut-level restriction [84,85]. Interestingly, *C. quinquefasciatus* mosquitoes naturally infected with OROV have been detected in field studies in Brazil [86], but their role as competent vectors remains unconfirmed.

In conclusion, the likelihood of sustained OROV transmission within European environments appears minimal under current conditions, largely due to the absence of competent vectors and ecological constraints [87,88]. However, given the rapid geographic expansion of OROV in the Americas, the confirmed vector competence of certain *Culicoides* species, and the increasing volume of intercontinental travel, a more comprehensive entomological evaluation—including the assessment of European midges—is warranted. Continued surveillance, laboratory-based vector competence studies, and climate-resilient preparedness strategies are essential to anticipate and mitigate any future risk of OROV emergence in Europe.

## 10. Italy and the Jubilee 2025

The Jubilee 2025, a major religious event traditionally held every 25 years, is expected to bring over 30 million pilgrims to Rome between December 2024 and January 2026, with additional arrivals following the recent death of Pope Francis [88]. A substantial portion of these visitors is expected from Latin America, a predominantly Catholic region and current epicenter of the Oropouche virus (OROV) epidemic [89,90].

Mass gatherings of this scale increase the risk of importing infectious diseases, particularly from areas with active transmission. The recent detection of OROV in two unrelated travelers returning from Cuba to Italy in May–June 2024 represents the first confirmed cases of OROV infection outside the Americas and highlights the virus’s potential for global dissemination [67,91]. Increased air traffic between Europe and Latin America, especially Cuba and Brazil, further amplifies this risk [90].

The ECDC has evaluated that, provided standard preventive strategies are implemented—including routine vaccination programs, appropriate hygiene measures, prompt self-isolation of symptomatic individuals, and safe sexual practices—the likelihood of exotic virus transmission, including OROV, to Jubilee participants remains low [76]. Historical precedent supports this assessment: during the previous Jubilee in 2000, which attracted approximately 26 million attendees, no significant increase in infectious disease cases was reported despite massive international gathering [92]. Nevertheless, while current entomological data suggest a low likelihood of autochthonous transmission—due to the absence of *Culicoides paraensis* in Europe and limited competence of *Aedes albopictus* and *Culex quinquefasciatus* [77,84]—knowledge gaps remain. In particular, the vector competence of European *Culicoides* species has not been systematically studied and could represent a potential risk under favorable ecological conditions [90].

## 11. Future Surveillance and Preparedness Recommendations

The emergence of OROV as a potential threat to European public health necessitates enhanced surveillance and preparedness measures. Future research efforts should expand vector competence assessments to include diverse European mosquito and biting midge populations, conduct multi-cycle transmission experiments under varying environmental conditions, and establish proactive surveillance systems as vector activity seasons progress.

Healthcare systems should maintain an awareness of OROV as a potential cause of febrile illness in travelers returning from endemic regions, particularly given the virus’s clinical similarity to other arboviral infections. Laboratory diagnostic capabilities should be enhanced to enable the rapid identification of OROV cases, and public health agencies should develop response protocols for potential importation events.

## 12. Conclusions

The 2024–2025 Oropouche virus outbreak has significantly expanded our understanding of this previously neglected arbovirus. The epidemic has been characterized by an unprecedented number of cases, broader geographic spread—including the first documented transmission in Cuba—and severe clinical outcomes such as neuroinvasive disease, hemorrhagic manifestations, and potential congenital complications. The emergence of reassortant strains with increased replication capacity and virulence highlights the virus’s evolutionary potential and underscores the need for continuous genomic surveillance.

While most OROV infections remain self-limiting, accumulating evidence suggests the possibility of vertical transmission and adverse pregnancy outcomes, including microcephaly. Although not yet definitive, these findings raise important concerns and justify further investigation. Despite legitimate concerns following imported cases in Europe, the risk of autochthonous transmission remains very low due to the absence of *Culicoides paraensis*, the primary vector in endemic areas, and the poor vector competence of European mosquito species. However, the role of native *Culicoides* species remains unknown and warrants urgent investigation, particularly in the context of climate change and increasing international travel.

Several critical knowledge gaps must be addressed. These include the mechanisms of neuroinvasion and vertical transmission, the genetic determinants driving increased virulence in reassortant strains, and the potential for sexual transmission and its public health implications. Further research is needed to identify risk factors for severe disease, better understand adverse pregnancy outcomes, and develop effective antiviral therapies. The availability of standardized, accessible diagnostic tools is essential to improve case detection, surveillance, and outbreak response.

## Data Availability

No new data were created or analyzed in this study. Data sharing is not applicable to this article.
